# Surgical Treatment of Madelung Deformity in Pediatric Patients

**DOI:** 10.7759/cureus.97208

**Published:** 2025-11-19

**Authors:** Soufiane Essamoud, Mohamed Lahlou, Achraf Benmazhar, Wassim Halli, Rania Bennani, Kenza Sekkat, Mohamed Abdel-Wahed, Hicham Zerhouni, Abdelmounim Cherqaoui

**Affiliations:** 1 Pediatric Surgery, Mohammed VI University of Sciences and Health, Casablanca, MAR; 2 Trauma and Orthopedics, Faculty of Medicine, Cairo University, Cairo, EGY; 3 Pediatric Orthopedic Surgery, Abderrahim Harouchi Mother-Child Hospital, Casablanca, MAR

**Keywords:** congenital wrist condition, léri-weill syndrome, madelung deformity, osteotomy, wrist deformity

## Abstract

Background

Madelung deformity is a rare congenital wrist condition characterized by the premature growth arrest of the distal radial physis, leading to wrist deformity and functional limitations. This study aimed to assess the short-term functional and radiological outcomes in patients with Madelung deformity treated surgically at two Moroccan hospitals.

Methods

This retrospective descriptive study included five skeletally immature patients (seven wrists) who underwent surgical treatment for Madelung deformity between January 2014 and July 2024. Patients underwent distal radial dome osteotomy, often combined with ulnar shortening osteotomy and Vickers ligament release. Outcomes were assessed using the Patient-Rated Wrist Evaluation (PRWE), Visual Analog Scale for pain and satisfaction, range of motion measurements, and radiographic parameters.

Results

The median follow-up was 39.2 months. Postoperatively, improvements were observed in most range of motion parameters, except in flexion. The median PRWE score improved (11/100 to 4/100), and patients reported high satisfaction with aesthetic outcomes. Radiographic parameters showed substantial median corrections in ulnar tilt (-12°; -28° to -4°), lunate fossa angle (-13°; -24° to 1°), lunate subsidence (-6 mm; -15 mm to -3 mm), palmar displacement (-11 mm; -14 mm to -8 mm), and palmar tilt (-29°; -50° to -28°). Two wrists required reoperation due to complications or insufficient correction. Two siblings with Léri-Weill syndrome demonstrated contrasting outcomes based on the timing of intervention and initial deformity severity.

Conclusion

Combined radial and ulnar osteotomies improved pain, function, and radiographic parameters in adolescents with symptomatic Madelung deformity in the short to medium term. Early diagnosis and intervention may lead to better outcomes. Larger, prospective studies with longer follow-up are needed to establish optimal treatment algorithms and long-term outcomes.

## Introduction

Madelung deformity is a rare congenital wrist condition first described by Otto Madelung in 1878 [1]. It is characterized by the premature growth arrest of the palmar and ulnar aspects of the distal radial physis, leading to increased palmar and radial tilt, carpal triangulation, lunate migration, and ulnar subsidence [2]. This deformity typically manifests in late adolescence and is more prevalent in females. Clinically, patients often present with wrist pain, restricted range of motion (ROM) (particularly extension and pronation-supination), and concerns regarding the aesthetic appearance of their wrists [3].

Surgical intervention may be considered when symptoms become progressive and have a significant impact on daily life [2]. Numerous surgical techniques have been proposed for the correction of Madelung deformity, including radial osteotomies (lengthening or corrective), ulnar osteotomies (shortening or epiphysiodesis), Vickers ligament release, and combinations of these procedures [2,4]. The goal of these surgical interventions is typically to reduce pain, improve ROM, correct deformities, and address aesthetic concerns [5,6]. Among the various surgical approaches, combined osteotomies of the radius and ulna are frequently employed to address the complex three-dimensional nature of the deformity and achieve more comprehensive correction [2,4,7].

Despite the variety of surgical options and satisfactory outcomes reported in many cases, the field lacks high-level evidence to guide treatment decisions. There is significant debate regarding the optimal surgical strategy [2,7]. Furthermore, the literature reports inconsistent postoperative outcomes, making direct comparisons between different surgical techniques and their results challenging. While many studies have reported pain reduction and improved ROM following surgery, it remains questionable whether current approaches consistently restore normal functional and radiological parameters. Achieving optimal short-term results is crucial, as these parameters form the necessary foundation for obtaining good long-term outcomes [6,8].

The primary aim of this study was to assess the short-term functional and radiological outcomes in a cohort of patients with Madelung deformity treated at our hospital as well as their impact on pain and patient satisfaction.

This article was previously posted to the Research Square preprint server on July 25, 2025 (DOI: https://doi.org/10.21203/rs.3.rs-7077906/v1).

## Materials and methods

Patients

This retrospective, observational, descriptive study included patients with Madelung deformity who were skeletally immature and were followed up and operated on between January 2014 and July 2024, at the Abderrahim Harouchi Mother-Child Hospital, Casablanca, Morocco. The exclusion criteria were patients who did not respond to the evaluation, with incomplete files, with skeletal maturity at the time of the intervention, and with less than 24 months of follow-up.

Data

**Figure 1:**
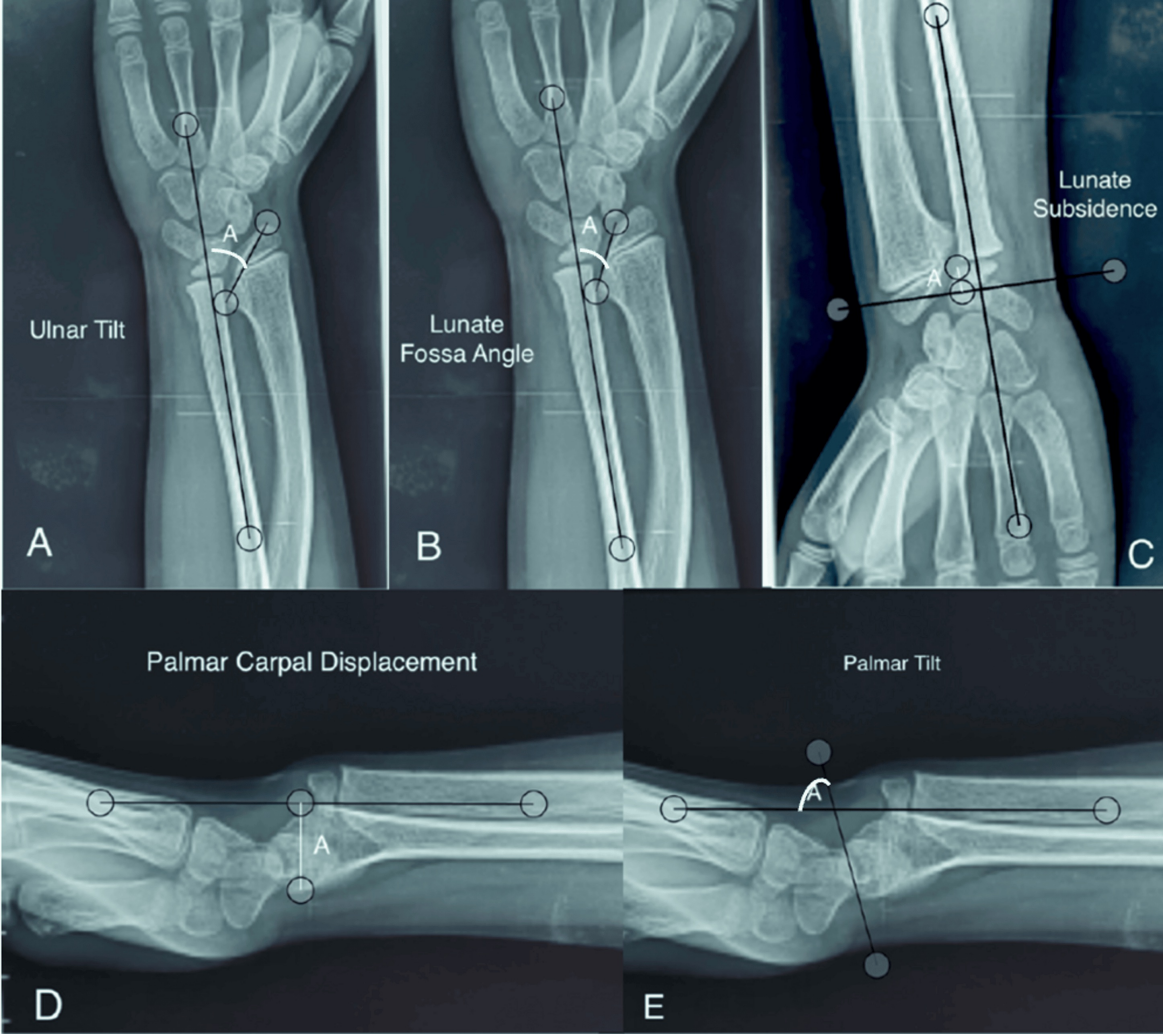
McCarroll et al.'s radiological criteria (A) Ulnar tilt: In the AP view, the ulnar tilt of the radial glenoid is defined as the complement (90°, angle A) of the acute angle A between the longitudinal axis of the ulna and the tangent of the radial articular surface of the scaphoid and lunate. It is greater than 33° in Madelung deformity. (B) Lunate fossa angle: On an AP view, the inclination of the lunate fossa is defined by the complementary acute angle (angle A) between the longitudinal axis of the ulna and the line along the lunate fossa of the radius. In Madelung deformity, it is greater than 40°. (C) Lunate subsidence: On an AP view, lunate subsidence is defined by the distance in millimeters between the most proximal point of the lunate and the line perpendicular to the longitudinal axis of the ulna at the distal articular surface of the ulna. In Madelung deformity, it is greater than 4 mm. (D) Palmar carpal displacement: On a profile view, palmar carpal displacement is defined by the distance in millimeters between the longitudinal axis of the ulna and the most palmar point of the lunate. In Madelung deformity, this is greater than 20 mm. (E) Palmar tilt: On a profile view, the palmar tilt of the radial glenoid is defined by the complement (90°, angle A) of the acute angle A between the longitudinal axis of the ulna and the line along the distal articular surface of the radius. AP: anteroposterior

After the study had been approved by the Ethics Committee of Centre Hospitalier Universitaire Ibn Rochd (approval number: CE-2025-113), the final evaluation was scheduled concurrently for all patients at the same time, and the following elements were collected, including standard demographic data such as age and sex, personal and family history, including history of hereditary disease in the family, similar symptomatology, and surgical intervention. Patient-reported outcomes were assessed using the Patient-Rated Wrist Evaluation (PRWE) questionnaire [9] and the Visual Analog Scale (VAS) for pain (0-10) and satisfaction (0-10). Pre- and postoperative measurements were compared to evaluate the surgical outcomes. Complications and reoperations were documented during the follow-up. The pre- and postoperative frontal and lateral wrist radiographs were analyzed and compared according to the five criteria of McCarroll et al. [10] at the last follow-up (Figure 1), in all patients: ulnar tilt (UT), lunate subsidence (LS), lunate fossa angle (LFA), palmar tilt (PT), and palmar carpal displacement (PCD). Radioulnar index (RUI), although not included in McCarroll et al.'s criteria, was valuable and was used in our study [11]. Occurrence or non-occurrence of complications and their nature, any re-do surgery, and postoperative patient satisfaction were also noted.

Operative technique

Patients underwent distal radial dome osteotomy (DRDO) [8] combined or not with ulnar shortening osteotomy (USO). DRDO was performed through Henry's anterior approach in the metaphyseal area. USO was performed via a posterior approach in case DRDO was not sufficient to address wrist deformity perioperatively. The Vickers ligament was systematically resected if identified. Fixation was performed using Kirschner wires and plates. Osteotomies and osteosynthesis spared the physis. The Sauvé-Kapandji procedure [12] was a "salvage" intervention for severe late-onset limitations in pronosupination. A screw was used to perform arthrodesis of the distal radioulnar joint. A long-arm cast was applied at the end of surgery.

Postoperative follow-up

The postoperative drains were removed after 24 hours. Healing of the osteotomies was observed six weeks later during a follow-up consultation, during which a forearm radiograph was performed, and the cast was removed following the confirmation of bone healing.

Data analysis

Clinical and radiological data were collected from medical records and stored in IBM SPSS Statistics for Windows, Version 27.0 (IBM Corp., Armonk, New York, United States). We conducted paired comparisons of preoperative and postoperative outcomes using the Wilcoxon signed-rank test. Continuous variables are summarized as medians with interquartile ranges (IQR). ROM and radiological parameters, as well as patient-reported outcomes, were analyzed as paired data within subjects. Two-sided tests were used, and effect sizes were derived from standardized test statistics when applicable. The follow-up duration was calculated from the date of surgery to April 20, 2025, and expressed in months.

## Results

Demographic profile of the cohort

We identified seven patients (10 wrists) who had been monitored and surgically treated for Madelung deformity. Five patients (seven wrists) matched our inclusion criteria, including four girls and one boy. The median age at the time of the consultation was 11.5 years (min=11; max=13 years).

Preoperative clinical and radiological assessment

Clinical Evaluation

The median duration from initial onset to first consultation was two years (range: 0-4 years), with one case being an incidental finding, while the remaining consultations were primarily for aesthetic concerns. There were four cases affecting the left wrist and three affecting the right wrist. Two cases of probable Léri-Weill syndrome were identified and were from the same sibling group, although genetic confirmation was not obtained. Pain was reported by three patients, and functional discomfort was noted in five, affecting all seven wrists. The median preoperative clinical ROM values indicated the characteristic limitations associated with Madelung deformity (Table 1).

**Table 1:** Clinical ROM: preoperative vs. postoperative ROM: range of motion; IQR: interquartile range

ROM parameters	Preoperative median (IQR)	Postoperative median (IQR)	Median improvement (IQR)	P-values
Pronation	90° (90° to 90°)	90° (89° to 90°)	0° (0° to 8°)	1.0
Supination	61° (60° to 75.5°)	83° (75.5° to 85.5°)	18° (-4.5° to 14.5°)	0.75
Flexion	80° (80° to 88°)	36° (18° to 53°)	-27° (-70° to -31°)	0.25
Extension	45° (10° to 45°)	30° (29° to 36°)	16° (6.5° to 30°)	0.5

Radiological Evaluation

The median preoperative radiological measurements are shown in Table 2.

**Table 2:** Radiological measurements: preoperative vs. postoperative

McCarroll et al.'s criteria	Measurement	Preoperative median (IQR)	Postoperative median (IQR)	Median correction (IQR)	P-values
	Ulnar tilt	52° (30.5° to 63°)	42° (32.8° to 51.3°)	-12° (-28° to -4°)	0.06
	Lunate fossa angle	74° (60° to 80°)	54° (51° to 61°)	-13° (-24° to 1°)	0.30
	Lunate subsidence	6 mm (5 mm to 14.5 mm)	2 mm (1.3 mm to 2 mm)	-6 mm (-15 mm to -3 mm)	<0.05
	Palmar displacement	26.5 mm (22.8 mm to 28.8 mm)	15 mm (14 mm to 15 mm)	-11 mm (-14 mm to -8 mm)	0.06
	Palmar tilt	19° (16.5° to 46.5°)	-11° (-12° to 0°)	-29° (-50° to -28°)	<0.05
	Radioulnar index	2 mm (1 mm to 6 mm)	0 mm (0 mm to 0 mm)	-2 mm (-6 mm to 0 mm)	0.25

Surgical treatment

All but three wrists underwent combined DRDO and USO. The Vickers ligament was identified and resected in six of the seven cases. One patient with bilateral condition underwent DRDO combined with USO on one side and the Sauvé-Kapandji procedure on the other. One patient underwent an isolated DRDO.

Postoperative results

The median follow-up duration for the cohort was 39.2 months, with an IQR of 31.9-44.5 months. The postoperative ROM measurements are shown in Table 1.

Surgical outcomes

Five operated wrists had satisfactory results, both functionally and aesthetically, with a reduction in exaggerated ulnar protrusion, an increase in joint amplitude, and a subjective increase in grip strength. Despite two wrists with unsatisfactory results, all of them reported that, given the choice once again, they would have opted for this procedure.

The patient-reported outcomes following surgery are presented in Table 3.

**Table 3:** Follow-up and functional outcomes: median (IQR) PRWE: Patient-Rated Wrist Evaluation; IQR: interquartile range

Outcome measure	Preoperative	Postoperative	P-values
Follow-up duration (months)	-	39.2 (31.9-44.5)	-
PRWE score	11 (6-36)	4 (2-19)	0.44
Pain score (0-10)	6.2 (4.4-6.8)	2.5 (2.5-2.8)	​<0.05​
Patient satisfaction (0-10)	-	9 (5-9)	-

Complications

Kirschner wires were removed upon bone consolidation in six wrists. The radial and ulnar dynamic compression plates were then retained. There were no occurrences of extensor tendon rupture, a complication frequently associated with Madelung deformity. Two wrists did not respond to treatment and would necessitate revision surgery: the wrist that underwent with great radial curvature and one wrist that presented pin infection that was removed before bone consolidation. Table 4 summarizes the individual findings of this cohort.

**Table 4:** Summary of the different individual findings in the study for each patient and wrist PRWE: Patient-Rated Wrist Evaluation; DRDO: distal radial dome osteotomy; USO: ulnar shortening osteotomy; VR: Vickers ligament release

Gender	Side	Time before the first consult	Main cause of consultation	Syndrome	Intervention	Preoperative/postoperative PRWE score	Global satisfaction (complication)
Patient 1 (girl)	Wrist 1	Fortuitous discovery	Fortuitous discovery	None	DRDO+VR	45/0	10/10
Patient 2 (girl)	Wrist 2	4 years	Aesthetics	None	DRDO+USO+VR	6/45	5/10 (pin infection)
Patient 3 (girl)	Wrist 3	1 year	Aesthetics	None	DRDO+USO+VR	5/2	8/10
	Wrist 4	1 year	Aesthetics	None	DRDO+USO+VR	8/4	8/10
Patient 4 (girl)	Wrist 5	3 years	Aesthetics	Léri-Weill	DRDO+USO	16/8	4/10 (surgical failure)
	Wrist 6	3 years	Aesthetics	Léri-Weill	Sauvé-Kapandji+VR	2/4	9/10
Patient 5 (boy)	Wrist 7	Family case	Family case	Léri-Weill	DRDO+VR	4/3	9/10

Leri-Weill syndrome

**Figure 2:**
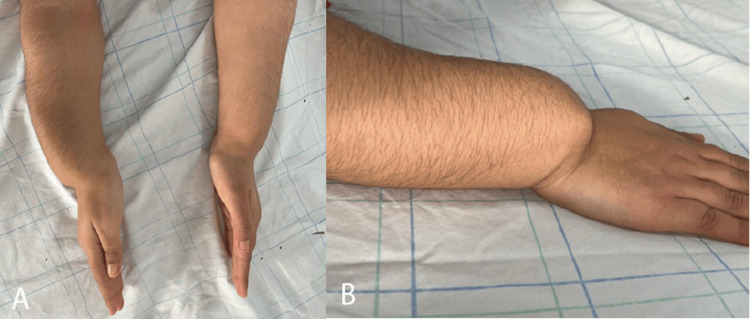
Clinical aspect of bilateral Madelung deformity in a girl suspected of having Léri-Weill syndrome: (A) top view and (B) lateral view

Two of the patients were siblings. The elder daughter first sought consultation at the age of 13 years, despite her deformity having been observed for four years in the right limb. She exhibited two arched wrists with posterior convexity and a significantly pronounced ulnar protrusion, accompanied by a marked limitation in joint amplitudes (Figure 2).

**Figure 3:**
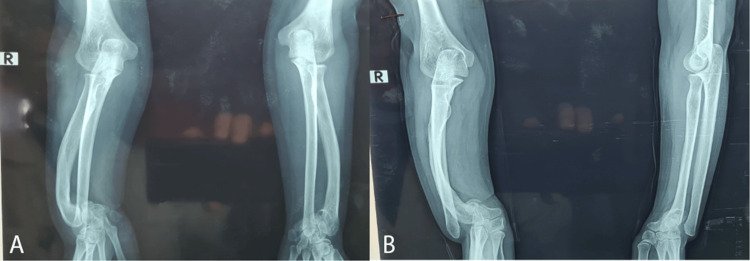
Radiographic images of both upper extremities: (A) anterior view and (B) lateral view

She displayed disharmonious dwarfism, which strongly suggested Léri-Weill dyschondrosteosis. No molecular or karyotype analysis was done because the family couldn't afford the testing. Radiologically, the right radius was severely deformed in the shape of an arch, with the radial glenoid looking anteriorly and the ulnar head dislocated posteriorly (Figure 3). The left side was less deformed.

**Figure 4:**
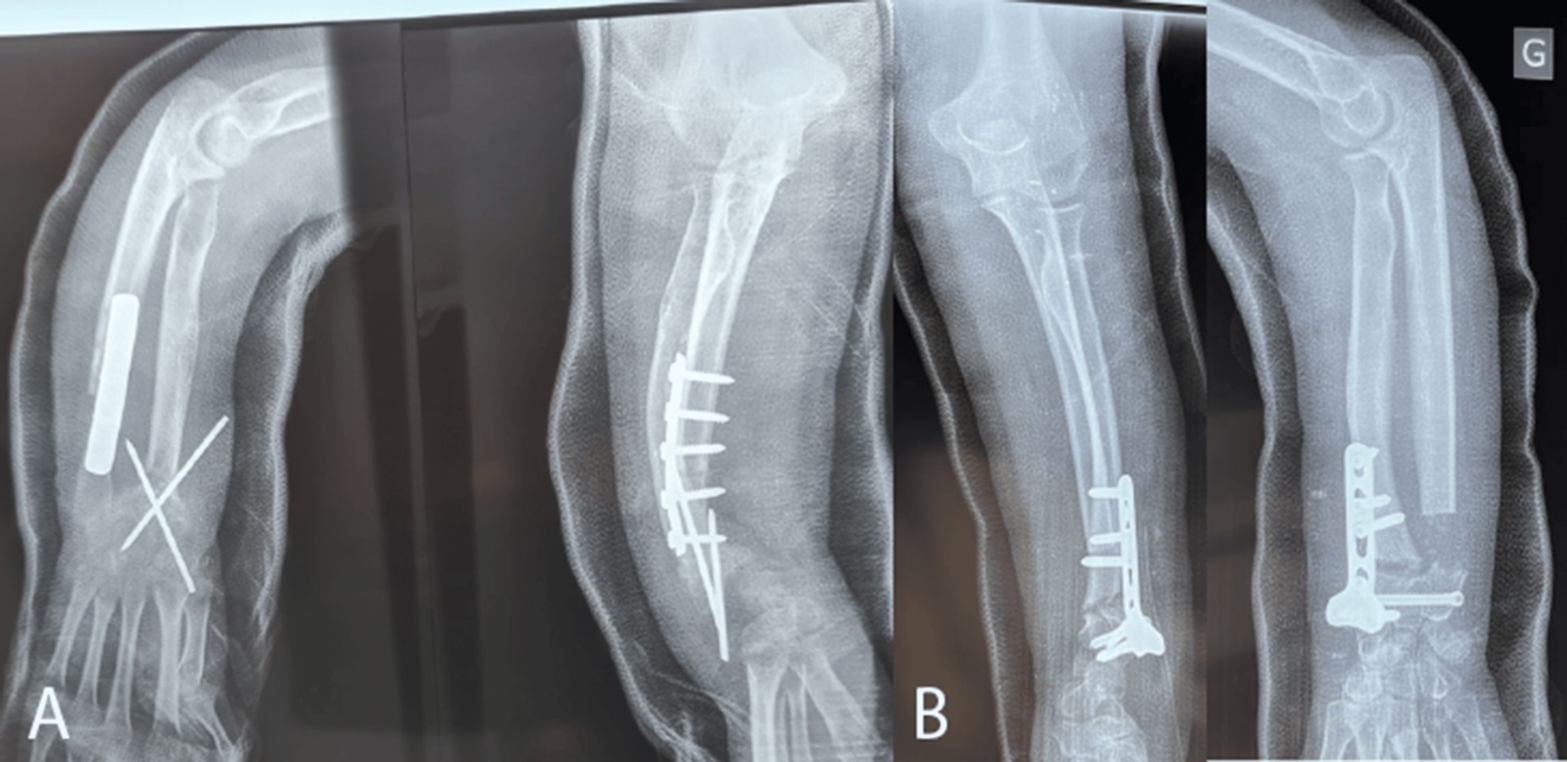
Postoperative radiographs of the forearms in the same patient: (A) right forearm and (B) left forearm

The right wrist underwent DRDO and USO, whereas the left forearm was treated with DRDO and the Sauvé-Kapandji procedure. Progression was further characterized by the unexpected consolidation of the ulna, which would necessitate resection in the future. The postoperative radiographs are presented in Figure 4.

Clinically, the right side, which exhibits a greater deformity than the contralateral side, is regarded as a therapeutic failure. Notably, joint amplitudes remained significantly restricted despite the intervention, with extension improvement, but at the cost of flexion (from 88° to 0°). Supination remained limited on the left side, likely due to the formation of a bony bridge.

**Figure 5:**
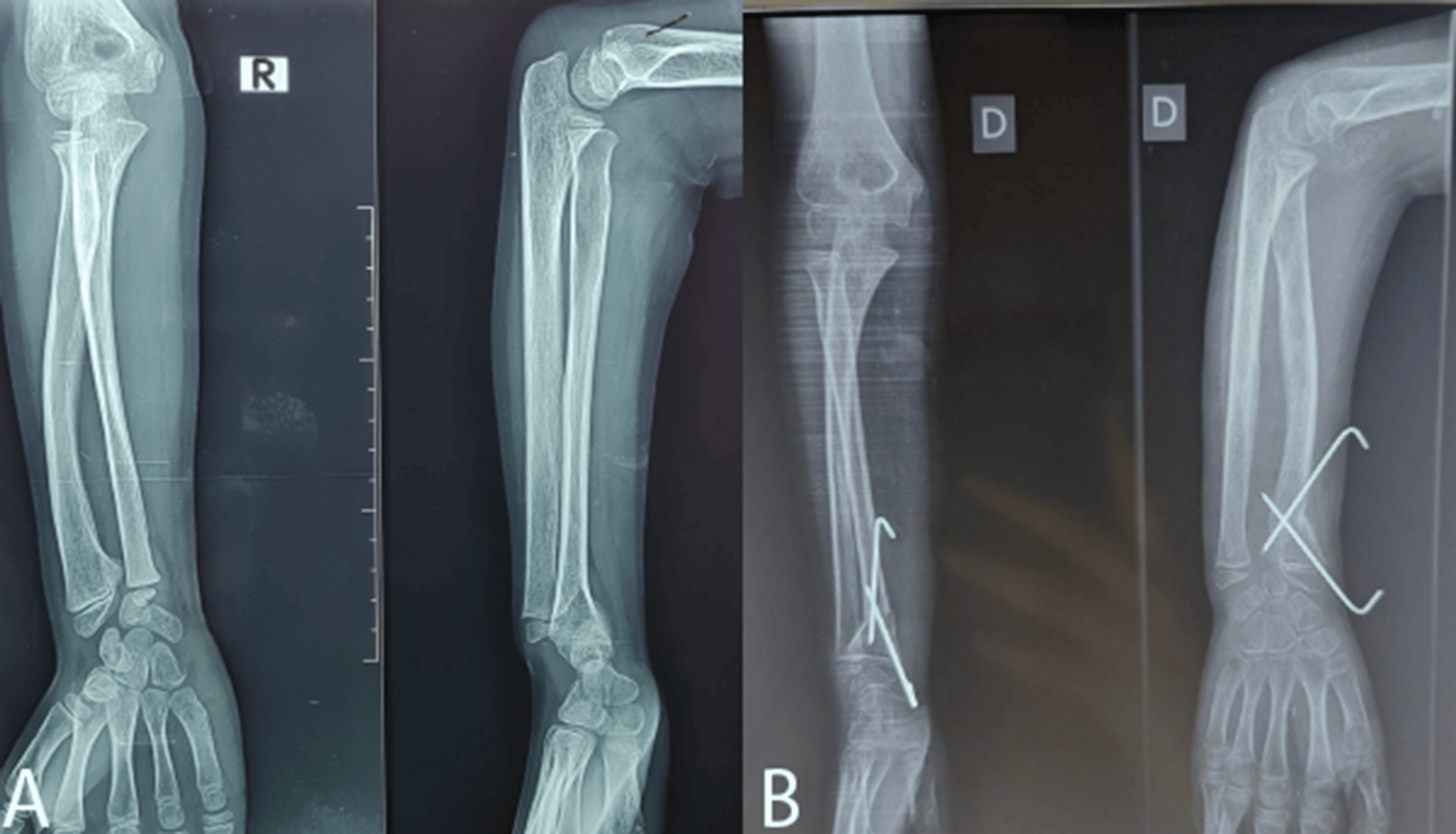
Wrist radiographs in the sibling with Leri-Weill syndrome: (A) preoperative radiographs and (B) postoperative radiographs

The brother, however, was diagnosed early, at the age of nine years, following parental recognition of the hereditary nature of the condition. He presented with a curvature at the lower end of the right radius, which was more pronounced on this side, without notable abnormalities in joint amplitudes. The patient underwent isolated DRDO with release of the Vickers ligament. Postoperatively, the joint amplitudes were within normal limits. Figure 5 shows the pre- and postoperative radiographs of the right wrist.

## Discussion

Madelung deformity, while uncommon, presents significant clinical challenges during adolescence, often prompting consultation owing to wrist pain, functional limitations, and aesthetic concerns [3,13,14]. Consistent with previous reports, our series found that aesthetic dissatisfaction was a primary driver for seeking medical attention, particularly among young female adolescents, often after a considerable delay from symptom onset (median: two years). This delay contrasts with some reports where pain was the predominant early symptom [15], but aligns with others, noting incidental findings or mild symptoms in younger children [16]. The underlying cause of pain remains debated, potentially stemming from Vickers ligament traction or altered joint biomechanics [15,17].

Our cohort exhibited characteristic ROM limitations associated with Madelung deformity, notably restricted extension, and, in our specific group, limited pronation, while supination was relatively preserved [18]. Postoperatively, we observed improvements in most ROM parameters, including pronation, supination, and, notably, extension, which often represents the most functionally limiting aspect. However, a significant decrease in the median flexion was noted. This can be attributed to the postoperative outcomes in one wrist (female Leri-Weill syndrome), which, considered a therapeutic failure, experienced loss of flexion. This substantial deficit in the range of flexion appears to be the cause of the decrease in the median flexion, as, after excluding this outlier patient, the median loss attenuated from roughly -52° to about -31°. Although subjective improvement in grip strength has been reported, the lack of objective pre- and postoperative dynamometer measurements, a limitation acknowledged later, prevents definitive conclusions regarding strength recovery [19].

Radiologically, preoperative measurements confirmed significant deformities according to McCarroll et al.'s criteria [10]. Postoperatively, substantial corrections were achieved in ulnar tilt, lunate fossa angle, lunate subsidence, and palmar carpal displacement (Table 2), consistent with the goals of surgical correction [5,6]. The median postoperative palmar tilt was negative, indicating an unintentional overcorrection. Given the significant remodeling potential known to exist at the distal radius in skeletally immature patients [20], this degree of correction was deemed acceptable and anticipated to remodel towards a more neutral alignment over time.

Advanced imaging techniques, such as 3D scanning or MRI, which are not routinely utilized in series due to resource constraints, offer potential advantages for precise preoperative planning and Vickers ligament assessment [21,22].

The surgical techniques employed, primarily DRDO combined with USO and Vickers ligament release, align with commonly reported approaches for moderate-to-severe deformities [2,4,7,10,15]. Isolated DRDO was performed in less severe cases, and the Sauvé-Kapandji procedure was used as a salvage option in one instance of severe late-presenting Léri-Weill syndrome. The frequent necessity for combined procedures (4/7 wrists) underscores the severity often encountered due to delayed presentation in our setting. Vickers ligament release was performed in almost all cases where it was identified, reflecting its perceived role in the pathogenesis [17]. Although isolated Vickers ligament release has been proposed for growth modulation in younger patients [1,15,17], it was not deemed sufficient for the established deformities in our adolescent series. Other techniques, such as gradual correction with external fixation (e.g., Ilizarov), have shown success, particularly in severe cases [23], but involve different management protocols and potential complications.

Our functional outcomes, assessed using the PRWE score and patient-reported pain, showed overall improvements, consistent with findings from other studies employing similar osteotomy techniques [8,14,24]. For instance, Leti Acciaro et al. [14] reported significant improvements in the Disabilities of the Arm, Shoulder, and Hand (DASH) score after modified dome osteotomy, and Harley et al. [8] noted pain reduction and ROM improvements after DRDO and Vickers ligament release. Carvalho et al. [24] also found good pain relief and functional scores post-DRDO. However, direct comparison across studies remains challenging due to variations in the outcome measures used (DASH, Mayo Wrist Score (MWS), PRWE), patient age, deformity severity, and follow-up duration. These inconsistencies likely contribute to the lack of definitive consensus on the single optimal surgical strategy noted in systematic reviews [2].

The inclusion of two siblings with Léri-Weill syndrome provides a compelling illustration of how the timing of intervention and initial deformity severity likely impact outcomes. Older siblings, who presented late with severe bilateral deformity, experienced a less favorable outcome on the more affected side despite surgical treatment. In contrast, the younger brother, who was diagnosed and treated earlier with a less severe deformity, achieved good functional results with isolated DRDO and Vickers ligament release. This aligns with the hypothesis supported by Guillaume et al. [7] in their long-term follow-up study that earlier intervention in less severe deformities yields better postoperative results.

This study had several significant limitations that must be acknowledged. First, the heterogeneity within our small cohort regarding the surgical procedures performed and the inclusion of syndromic cases makes it difficult to isolate the effects of specific interventions. In addition, the median follow-up duration of approximately three years, while providing valuable short- to medium-term data, is insufficient to assess long-term outcomes, such as the development of osteoarthritis or the durability of the correction, particularly concerning the remaining growth potential. As previously mentioned, the absence of objective grip strength measurements limits functional assessment. Finally, potential biases inherent to retrospective data collection may exist.

## Conclusions

Despite these limitations, our findings support the efficacy of combined radial and ulnar osteotomies, including DRDO, in improving pain, function (as measured by PRWE), and radiographic parameters in adolescents with symptomatic Madelung deformity in the short to medium terms. Patient satisfaction, particularly with regard to aesthetic improvement, was high. The contrasting outcomes in siblings with Léri-Weill syndrome underscore the potential importance of early diagnosis and intervention before the deformity becomes severe. Future research should ideally require prospective, multicenter studies with larger cohorts, standardized protocols, objective functional measures, and longer follow-up periods to definitively establish optimal treatment algorithms and long-term outcomes.
